# Supplementary material for: Inhibition of Angiogenesis and Effect on Inflammatory Bowel Disease of Ginsenoside Rg3-Loaded Thermosensitive Hydrogel
Source: Pharmaceutics. 2024 Sep 25;16(10):1243. doi: 10.3390/pharmaceutics16101243 (PMC11509886; doi:10.3390/pharmaceutics16101243)
Supplement: Supplementary file 1 [file pharmaceutics-16-01243-s001.zip › pharmaceutics-3137017-supplementary.pdf]

Supplementary Materials

1. The calibration curve of the HPLC method was shown in Figure S1, and the concentration range for Rg3 was 0.5045-50.4465 μg/mL.

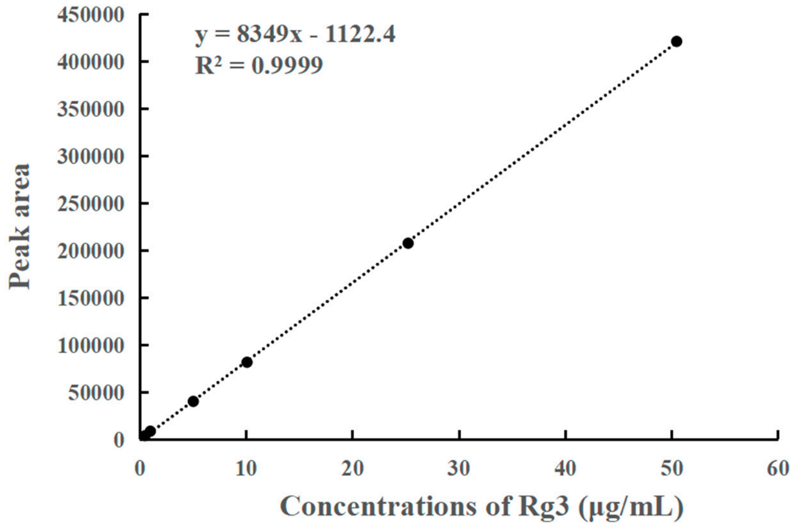


Figure S1. Calibration curve of Rg3.
